# Additional intramuscular iron injections tended to improve post-weaning growth when administered at weaning, but not at day seven of life

**DOI:** 10.1093/tas/txaf133

**Published:** 2025-10-10

**Authors:** James E Langley, Kate J Plush, Surinder S Chauhan, John R Pluske, Frank R Dunshea, Jeremy J Cottrell

**Affiliations:** School of Agriculture, Food, and Ecosystem Sciences, Faculty of Science, The University of Melbourne, Parkville, Victoria, 3052, Australia; SunPork Group, Eagle Farm, Queensland, 4009, Australia; School of Agriculture, Food, and Ecosystem Sciences, Faculty of Science, The University of Melbourne, Parkville, Victoria, 3052, Australia; School of Agriculture, Food, and Ecosystem Sciences, Faculty of Science, The University of Melbourne, Parkville, Victoria, 3052, Australia; School of Agriculture, Food, and Ecosystem Sciences, Faculty of Science, The University of Melbourne, Parkville, Victoria, 3052, Australia; Faculty of Biological Sciences, University of Leeds, Leeds, LS2 9JT, United Kingdom; School of Agriculture, Food, and Ecosystem Sciences, Faculty of Science, The University of Melbourne, Parkville, Victoria, 3052, Australia

**Keywords:** anemia, growth, health, iron, pig, weaning

## Abstract

Piglets have a high requirement for iron due to their rapid growth rates and low body iron stores; moreover, intensive production conditions restrict access to environmental sources of iron, such as soil. The low iron content of sow colostrum and milk, combined with the partitioning of limited nutrients among many piglets in a litter, results in inadequate piglet iron intake, predisposing them to iron-deficient anemia (IDA) during lactation and weaning. Prevalent IDA results in reduced post-weaning growth and reduced hemoglobin (Hb) concentrations. To combat this, indoor-reared piglets are given an injectable iron supplement shortly after birth to maintain body iron stores until weaning, when feed containing dietary iron is consumed. Due to continued selection for growth rates and litter sizes, this single injection is now considered inadequate to meet iron requirements. It was hypothesized that an additional iron injection, irrespective of timing, would improve Hb concentrations and growth performance, with a day 7 injection resulting in higher Hb and growth at weaning and a weaning injection increasing Hb and performance in pigs later in the nursery period. A total of 440 mixed sex pigs were used in this experiment in three treatment groups: control: only receiving iron dextran two days after birth; day 7: receiving iron injections at days two and seven after birth; Weaning iron: receiving iron dextran injections on day two and at weaning (20.1 days). Hb concentrations were significantly (*P *< 0.001) improved in the day 7 treatment for 18 days after weaning. Pigs in the control and weaning iron groups had a treatment mean of 9.9 g/dL ± 0.32 and 9.7 g/dL ± 0.32, respectively, indicating sub-clinical anemia, compared to the day 7 group with a treatment mean of 12.2 g/dL ± 0.32, indicating healthy Hb concentrations. Average daily gain tended (*P* = 0.08) to be higher in the weaning iron treatment group during the second week of the experiment compared to the control and day 7 groups. Pig removals were also significantly (*P* < 0.001) reduced in the weaning treatment group. Plasma hepcidin anti-microbial peptide, was elevated in the weaning iron group after injection, possibly impairing enteric iron absorption. An additional injection at weaning showed potential for improved growth in the post-weaning period and has practical advantages for producers.

## Teaser

An additional iron injection at weaning showed a strong trend for increased growth in the second week after weaning.

## Introduction

Iron fulfils numerous essential roles in the body, including serving as a carrier of molecular oxygen, being involved in the electron transport chain, and DNA synthesis (Kim et al 2018). Blood represents in excess of 70% of the total iron stores, principally in the form of hemoglobin (Hb) (Almond et al 2017). Piglets are born with approximately 50 mg of iron(Chevalier et al 2021), and their daily requirements are determined to be in the range of 8 to 16 mg, with levels below this increasing the risk of anemia. Sow colostrum and milk are poor sources of iron (1.22 mg/L), and when combined with total milk produced by a sow being shared between all piglets in the litter, piglets cannot meet their iron requirements by suckling alone (Pond et al 1965).

Reduced iron status has been linked to reduced feed intake (Knight and Dilger 2018) and therefore anemic pigs cannot grow as fast as healthy iron status pigs. Currently, Hb is used as the most prominent marker of iron-deficient anemia (IDA) in pigs, and point-of-care meters such as the Hemocue are fast and effective tools that can be used to rapidly quantify Hb status within a herd. The current classification of anemia is <9 g/dL clinical anemia, 9 to 11 g/dL; subclinical anemia and >11 g/dL healthy iron status (Bhattarai and Nielsen 2015a). This value does not account for any other iron stores in the body that could be measured, such as total iron-binding capacity or ferritin, an important variable in human medicine in the diagnosis of IDA. Nevertheless, using these values, IDA has been widely reported in pigs globally, including in Australia. In a Canadian study, 42% of pigs weaned were sub-clinically or clinically anemic (Perri et al 2016), with rates in Australia following similar trends for sub-clinical anemia at weaning (Hewitt and Zemitis 2021). The impact of IDA is a reduction in animal health and reduced growth rates associated with disease and reduced feed intake during the post-weaning period (Bhattarai and Nielsen 2015a; Knight and Dilger 2018). For this reason, Beta Hydroxybutyrate (BHB) was measured as it can be used as an indicator of ketosis, associated with reduced feed intake, and muscle wasting in the early post-weaning period (Perri et al 2016).

The relationship between Hb and post-weaning weight gain has been previously examined in Europe but, not yet been examined in Australia, with results showing an increase of 1 g/dL, increasing weight gain by 17.2 g/d (Bhattarai and Nielsen 2015a). However, when a similar study was performed in Canada, this relationship could not be established (Seip et al 2020) plausibly highlighting genetic differences in pigs internationally. This is especially relevant to the closed Australian pig herd, as due to genetic isolation, Australian pigs differ significantly in certain areas, such as paler meat color (Ngapo et al 2007), a factor that has been shown to be influenced by iron status (Dunshea et al 2024) potentially reducing Australian pigs requirements for iron.

Iron dextran, an organic, glucose-based iron supplement, is typically given to indoor-born pigs within 1 to 2 days of farrowing via intramuscular injection. This dose is approximately 200 mg of elemental iron and, there has been success in reducing IDA, and increasing growth by adjusting the injection regime with an additional injection at day 7 after farrowing (Pierce et al 2024). But the effect of an additional iron injection is yet to be quantified in Australia. One potential issue with supplementation at weaning is the impact of high extracellular iron on hepcidin, the key hormone in the absorption of dietary iron. There is a possibility that hepcidin could be elevated at the time of weaning, potentially impairing enteric iron absorption, as shown (Starzyński et al 2013).

Therefore, the aim of this study was to apply the previous knowledge gained from additional iron supplementation of pigs at day 7 to an additional iron injection at weaning to examine if growth and Hb improvements can still be observed in a weaning injection in the first four weeks after weaning in Australian pigs.

## Materials and methods

Approval for conducting this study was granted from CHM Alliance Pty Ltd Animal Ethics committee (CHM PP 167/23), and the study was conducted in accordance with the Australian code for the care and use of animals for scientific purposes 8^th^ edition 2012. (National Health and Medical Research Council 2013).

### Animals and housing

The experiment was conducted at a commercial research piggery in Queensland, Australia and consisted of 33 pens of 14 pigs per pen, with litters assigned a treatment at piglet processing (day 2 of life). Piglets were weaned at 20.2 ± 0.07 days into the experimental nursery in four replicates, separated by sex, balanced for weight and housed with a spacing allowance of 0.20 m^2^/pig on fully slatted flooring. All pigs were fed the same standard starter diet (Table 1) with tested iron levels of 180 mg/kg of feed for the duration of the nursery period. Feed was provided by a circular gravity feeder in the center of each pen, and water was available through one bowl and one nipple drinker in each pen.

**Table 1.** Dietary formulation (%) as fed and near infrared spectroscopy analysis of diet fed to pigs for the entire trial period.

Ingredient	
**Soybean Meal**	22.0
**Wheat**	51.6
**Biscuit meal sweet**	5.00
**Soya**	4.95
**Soya bean**	8.00
**Blood meal**	2.00
**Meat meal**	2.95
**Fish meal**	5.00
**Whey powder 11.5 %**	12.5
**Hilyses**	2.00
**Bovacillus**	0.03
**Canola oil**	1.95
**Mono dical phosphate**	0.25
**Salt**	0.20
**Zinco plus**	0.10
**Betaine**	0.10
**DL-methionine**	0.16
**Lysine HCL**	0.40
**L-threonine**	0.11
**L-tryptophan**	0.03
**L-isoleucine**	0.01
**Rovabio advance P 10%**	0.05
**HI-PHOS Phos starter diets**	0.008
**Activo**	0.02
**Fysal**	0.40
**Krave**	0.03
**Starter premix**	0.20
**Calculated composition**	
**DE Pigs (MJ/kg)**	14.8
**SID Lysine (%)**	1.34
**Crude Protein %**	22.6
**SID Lys: ME**	0.09
**SID Lysine: CP**	0.06
**Iron mg/kg**	180

Starter premix contains: Vit A 10 miu, Vit D3 2 miu, Vit E 80 ppm, Vit K 3 ppm, Vit B1 3 ppm, Vit B2 4 ppm, Vit B6 3 ppm, Vit B12 20 ppm, Pantothenate 20 ppm, Niacin 25 ppm, Biotin 10 ppm, Folic 0.5 ppm, Fe TOTAL 70 ppm, Zn TOTAL 100 ppm, Mn TOTAL 40 ppm, Cu 20 ppm TOTAL, Se TOTAL 0.1 ppm.

The standard on-farm medication regime was followed during the nursery phase of the experiment. This comprised a water acidifier (1% Selko, Trouw Nutrition, Amersfoort, Netherlands) for all pigs from entry to day seven after weaning. On days 12 to 14, a preventative antibiotic was administered via water (Oxytetracycline 25 mg/L). On day 25, piglets received two vaccinations, for prevention of *Mycoplasma hyopneumoniae* infection (M-Pac, MSD animal health, USA) and Leptospirosis (Eryvac, Zoetis, Australia).

### Treatments

Litters were randomly assigned to a treatment group from 141 sows in parities 2 to 6 at piglet processing. On day two, pigs were given an individual ear tag with a unique number used for identification during the experiment. During this time, all pigs received the first iron dextran injection using a 21G needle into the muscle directly behind the ear (1 mL Ferron 200 + B_12_ that included 40 µg/mL vitamin B_12_ as cyanocobalamin; Ferron, Elanco, IN, USA). During this time, piglets also had tails docked and were administered an oral coccidiostat (Baycox Coccidiostat, Elanco Australia, Macquarie Park, New South Wales, Australia). At seven days of age, pigs in the day 7 group were given an additional dose of Ferron 200 + B_12_. At weaning, all pigs were then moved to the nursery facility, where the weaning treatment group was administered the additional Ferron 200 + B_12_.

### Measurements

*Nursery performance*. Pigs were weighed weekly from entry until four weeks after weaning, with individual data summed to pen growth for each period. The amount of feed offered and residuals were measured every week and assumed to be pen feed intake. Pen growth rate and feed intake data were used to calculate the gain: feed ratio on a pen basis. Any pig requiring medical intervention was recorded (cause and treatment) and removed to a hospital pen (removals). A pig was only removed if it was deemed unable to survive without treatment.

*Behavioral analysis*. Pigs were recorded using video cameras suspended above pens (Swann 4-channel wireless Wi-Fi NVR package, Melbourne, Australia) from 0700 to 0900 h on days four to seven after weaning. Behaviors were recorded against the following ethogram (Table 2) using the scan sampling technique with a sampling interval of 30 seconds. Scan sampling is known to be an ideal way to assess the behavior, posture and activity levels of pigs, with closer sampling periods providing more accurate data (Kobek-Kjeldager et al 2024). For every sample, each pig within the pens was scored for both posture and activity.

**Table 2.** Ethogram of behaviors for observations of pigs in this behavior study. Animals were observed from days four to seven after weaning from 0700 to 0900 h. Pigs were scan sampled every 30 seconds.

Activity	
**Eating**	Piglets eating from the feeder or chewing feed
**Drinking**	Piglet touching or drinking from the nipple or bowl drinker.
**Active**	Piglet active, performing behaviour not characterized in this ethogram.
**Champing**	Masticating without substrate in the mouth (Pastorelli, 2012)
**Inactive**	Piglet is lying or sitting and may be asleep.
Posture	
**Sitting inactive (dog-sitting)**	Sitting with hind legs stretched forward under the body
**Lying**	Laying down on belly or side
**Sick Pig stance**	Standing still head down may or may not be champing, ears forward. (Perri et al 2016)
**Standing**	Standing on all four legs, may or may not be moving.

*Blood collection*. On days one and 28 of weaning, four focal pigs per pen were identified and had a blood sample collected via jugular venipuncture using a 21G needle into EDTA vacutainers. The timing of sampling occurred two hours after administration of iron dextran and was standardized across days. Following collection, samples were placed in a centrifuge and spun for ten minutes at 1000 g, with plasma extracted and stored at −20 °C until analyses.

*Whole blood measurements*. On days zero, four, seven, 11, 14, 18, 25 and 28 after weaning, the same four pigs were sampled for Hb (HB201+ blood analyzer, Haemocue AB, Sweden) and BHB concentrations (Freestyle Optimum Neo Blood Ketone Meter, Abbott Industries, Chicago, United States) from each pen (*n* = 44 per treatment). This was done by collecting a small amount of blood from the ear using a fine needle directly into a cuvette and following the supplier’s instructions. The haemocue Hb201+ was validated for use as an accurate point-of-care meter for Hb quantification (Kutter et al 2012). The BHB meter was validated for use in cattle (Sailer et al 2018), as BHB is an indicator of ketosis and wasting in pigs in the post-weaning period (Perri et al 2016).

*ELISA analyses*. Plasma collected on the farm was analyzed using ELISA for the concentrations of haptoglobin (ABClonal, Boston, Massachusetts, United States), ferritin (Cloud Clone, Houston, Texas, United States) and HAMP (Cloud Clone). The spike recoveries for haptoglobin, ferritin and HAMP were 104%, 90% and 117% respectively, and the intra-assay variability was 8%, 4% and 6%, respectively.

*Statistics*. Pig production data were analyzed by analysis of variance with treatment as a fixed effect and pen as a blocking factor using GenStat, version 23 (Rothamsted Research, England, United Kingdom). The pig behavioral data did not meet normality assumptions and were analyzed in SPSS (IBM Corp. Released 2023. IBM SPSS Statistics for Windows, Version 29.0.2.0 Armonk, NY: IBM Corp) using negative binomial regression with a repeated measure of day. As not all pens contained the same number of pigs (removals), the number of pigs in each pen was included as a covariate. The model included the fixed terms of day, treatment and the interaction between day and treatment. The ELISA results did not meet normality tests using any transformation. Therefore, analysis of variance was used to generate means, separated using non-parametric Kruskal-Wallis testing with treatment as the fixed effect in SPSS. Statistical significance was accepted with a *P* value of < 0.05, and a trend was *P* value < 0.1.

## Results

### Pen-side blood parameters (Hb and BHB)

**Fig. 1.**
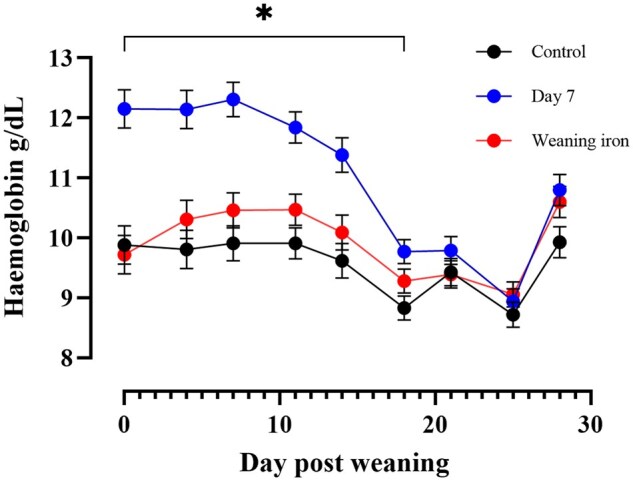
Hemoglobin (Hb g/dL) concentration (mean ± SEM) for 28 days after weaning for pigs on a treatment basis given different iron treatments. The control group was given 200 mg of iron on day two after farrowing. The day 7 group was given control and 200 mg iron dextran on day seven after farrowing and weaning iron was given control and 200 mg iron dextran at weaning. Data was analyzed via analysis of variance. *denotes *P* < 0.05 from control for day 7 group.

The Hb levels were significantly higher in the day 7 treatment group until day 18, after which point all treatments were similar (Fig. 1).

**Fig. 2.**
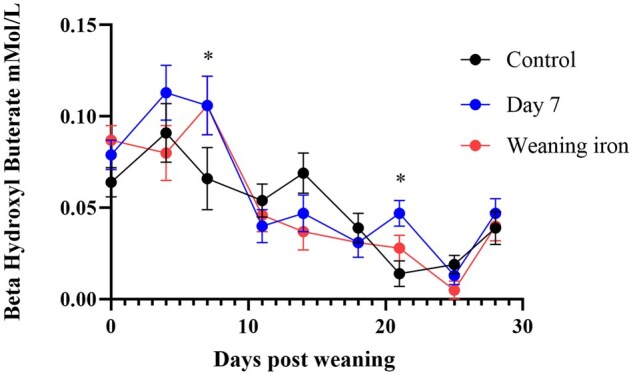
Beta-hydroxyl butyrate (BHB) concentrations for 28 days after weaning for pigs given different iron treatments. The control group was given 200 mg of iron dextran two days after farrowing. The day 7 group was given control and 200 mg iron dextran on day seven after farrowing and weaning iron was given control and 200 mg iron dextran at weaning. * indicates *P* < 0.05 from control.

There was a trend for elevated BHB concentrations in both treatment groups on day seven after weaning (*P* = 0.1) and in the day 7 treatment group on day 21 after weaning (Fig. 2). There was a significant effect of time (*P* < 0.05) on all treatment groups, with BHB concentrations decreasing over time.

### Behavioral analyses

Treatment had an impact on activity, weaning iron pigs were found to be inactive (sitting and lying) more frequently, followed by day 7, with the control group exhibiting the lowest frequency for this behavior (Table 3). There was no difference between treatments in eating, drinking or champing behaviors, with overall frequency of these behaviors being low (8.8%, 0.91% and 0.18% of total observations, respectively). The impact of day was more pronounced, influencing both posture (lying, standing) and activity (active, inactive and drinking). When averaged across all measurement days, pigs from the weaning iron treatment were observed to be lying more frequently, and control pigs were observed to stand more frequently, with the highest period of activity on day four after weaning. There was no significant effect of the interaction between day and treatment. There was no difference in the sick pig stance between the three treatments.

**Table 3.** Frequency of behaviors (120 observations each day for four days) performed by pigs given different iron doses and timings between 0700 and 0900 h on days four-seven after weaning, (mean ± SEM shown). A total of 5280 observations were recorded for treatment and 1320 for each day were recorded. Pigs were scan sampled every 30 seconds. The control group was given 200 mg of iron on day two after farrowing. The day 7 group was given control and 200 mg iron dextran on day seven after farrowing and weaning iron was given control and 200 mg iron dextran at weaning.

Frequency	Treatment			Day				*P* value		
	Control	Day 7	Weaning iron	4	5	6	7	Treatment	Day	D*T
**Posture**										
**Lying**	1092 ± 76^a^	1190 ± 87^a^	1378 ± 83^b^	806 ± 495^a^	1313 ± 515^b^	1440 ± 546^b^	1341 ± 369^b^	0.032	0.001	0.99
**Standing**	2168 ± 78^a^	1981 ± 91^b^	1941 ± 84^b^	2330 ± 602^a^	1977 ± 524^ab^	1852 ± 563^ab^	1949 ± 382^b^	0.053	0.013	0.97
**Sitting**	13.75 ± 1.1	11.1 ± 1.4	11.2 ± 0.9	11.3 ± 7.5	11.4 ± 6.1	11.6 ± 4.9	13.6 ± 10	0.20	0.74	0.62
**Sick Pig Stance**	15.5 ± 1.6	16.3 ± 1.2	15.9 ± 1.4	16.5 ± 10	16.9 ± 9.2	15.1 ± 7.7	14.9 ± 8.3	0.91	0.78	0.99
**Activity**										
**Active**	1637 ± 64	1466 ± 75	1454 ± 69	1779 ± 522^a^	1520 ± 419^ab^	1328 ± 440^b^	1442 ± 285^b^	0.06	0.002	0.98
**Inactive**	1109 ± 76^a^	1208 ± 87^b^	1389 ± 83^c^	819 ± 494^a^	1330 ± 511^b^	1451 ± 543^b^	1361 ± 364^b^	0.047	<0.001	0.99
**Eating**	488 ± 22	459 ± 22	447 ± 21	507 ± 144	420 ± 140	467 ± 157	462 ± 114	0.21	0.15	0.82
**Drinking**	45.3 ± 3.9	54.5 ± 3.7	44.7 ± 5.1	50.5 ± 18^ab^	37.1 ± 14.7^a^	62.7 ± 29.0^b^	41.8 ± 37.0^ab^	0.22	<0.001	0.77
**Champing**	9.3 ± 1.3	10.4 ± 1.9	9.91 ± 1.2	7.74 ± 5.8	11.0 ± 9.1	9.35 ± 6.7	11.3 ± 8.4	0.12	0.55	0.83

a–c Indicates statistical significance.

### Growth performance

The growth rate, feed intake and production efficiency data (Table 4) were not different between treatments, although there was a tendency (*P* < 0.10) for improved ADG and gain: feed in week two in the weaning iron treatment group. There was a statistical difference in removals with less pigs removed from the weaning iron treatment group.

**Table 4.** Post-weaning feed intake and piglet growth performance (mean ± SED). The control group was given 200 mg of iron on day two after farrowing. Day 7 group was given control and 200 mg iron dextran on day seven after farrowing and weaning iron was given control and 200 mg iron dextran at weaning. Data was analyzed using analysis of variance with pen as the experimental unit.

**Parameter** ^a^	Treatment			SED	*P* value
	Control	Day 7	Weaning iron		
**Nursery (overall)**					
**ADFI, g/pig**	377	381	389	11.5	0.50
**ADG, g/pig**	272	270	289	12.8	0.30
**Gain: Feed**	0.65	0.67	0.69	0.022	0.40
**Weaning weight, Kg**	6.31	6.46	6.46	0.158	0.55
**Week 1**					
**Weight Kg**	6.60	6.79	6.74	0.164	0.49
**ADFI, g/pig**	115	129	116	8.6	0.22
**ADG, g/pig**	34.9	53.2	42.0	11.38	0.30
**Gain: Feed**	0.29	0.42	0.36	0.097	0.43
**Week 2**					
**Weight, Kg**	8.31	8.52	8.61	0.201	0.30
**ADFI, g/pig**	277	279	286	13.3	0.76
**ADG, g/pig**	237	235	268	16.2	0.080
**Gain: Feed**	0.86	0.84	0.94	0.049	0.090
**Week 3**					
**Weight, Kg**	10.6	10.8	11.1	0.25	0.162
**ADFI, g/pig**	469	466	483	19.9	0.76
**ADG, g/pig**	318	314	355	28.6	0.28
**Gain: Feed**	0.68	0.67	0.74	0.048	0.30
**Week 4**					
**Weight, Kg**	14.1	14.3	14.5	0.33	0.51
**ADFI, g/pig**	661	657	675	19.9	0.69
**ADG, g/pig**	501	481	491	35.9	0.87
**Gain: Feed**	0.76	0.74	0.73	0.051	0.83
**Number of pigs removed from the trial**	5^b^	6^b^	0^c^		<0.001

ADFI, average daily feed intake; ADG, average daily gain.

a–c Indicates statistical difference.

**Fig. 3.**
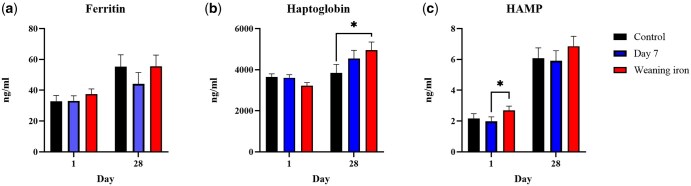
a) Ferritin, b) haptoglobin and c) hepcidin anti-microbial protein (HAMP) results (mean ± SED). Blood samples were collected at weaning and 28 days after via jugular venipuncture. The control group was injected 200 mg of iron dextran two days after farrowing, while the day 7 and weaning iron groups were given control and an additional 200 mg at day 7 after farrowing or at weaning. ^a–c^indicates statistical significance (*P* < 0.05). Data was not normally distributed, analysis of variance was used to generate means, and non-parametric Kruskal-Wallis testing was used to separate means. As a normal distribution could not be achieved, no interaction *P*-value exists.

*Plasma ELISA analyses*. An increase in HAMP was identified in the weaning iron treatment group (*P* < 0.05) when compared to the day 7 treatment group (Fig. 3). There was also a significant increase in the haptoglobin concentrations of the weaning iron treatment group compared to the control group on day 28.

## Discussion

The results of this experiment showed that an additional iron injection at weaning resulted in trends for improved growth in the second week after weaning. It was hypothesized that the day 7 injection would improve Hb and growth at weaning as previously shown in literature, and that the weaning iron treatment would provide benefit later in the nursery period. Whilst the Day 7 treatment was successful at improving Hb during the nursery period, the Weaning group had similar Hb concentrations to those of the Control pigs. Despite this, a trend for increased ADG and gain: feed in the second week after weaning was observed in the weaning pens. The experiment also confirmed that large numbers of pigs were weaned with sub-clinical anemia, but not clinical anemia, this is possibly a result of sow iron status influencing piglet Hb. There were also significantly less removals in the weaning treatment group, possibly indicating a weaning iron injection has implications for animal health. Furthermore, HAMP concentrations were significantly elevated in the Weaning treatment, which may suppress enteral uptake of iron, helping to explain these contrasting results, consistent with results published in Europe (Starzyński et al 2013).

In this experiment, Hb was assumed to be a reliable marker of iron status, as is commonplace in veterinary and human medicine. However, the evolving understanding of iron metabolism and the turnover of red blood cells may no longer support this assumption, as Hb concentrations are affected by more factors than just the availability of iron for erythropoiesis. It is possible that for this reason, Hb concentrations did not improve in the same way as the day 7 injection later in the weaning period, but a trend for increased growth was observed in the weaning iron treatment group in the second week after weaning (ADG and gain: feed). Another possible explanation for the lack of increase in Hb is the change in dosage size pigs received based on different ages. All pigs received approximately 131 mg/Kg bodyweight of iron at day two after farrowing. This decreases to 75.4 mg/Kg bodyweight for day 7 pigs and weaning pigs receiving 31.3 mg/kg liveweight at weaning (data not shown). As the day 7 injection was the only treatment to have an effect of Hb, it is reasonable to assume that a dosage of 75.4 mg/Kg is the minimum effective dose to affect Hb in pigs.

Clinical parameters of IDA in pigs are poorly defined as a species, let alone in differing genotypes or production systems. There have been several studies that classify clinical anemia as Hb concentrations < 9 g/dL (Bhattarai and Nielsen 2015b; Estienne et al 2019; Thorn et al 2022) in pigs. However, other papers classify IDA at 10 g/dL (Prunier et al 2022; Menegatt et al 2025) or lower at 8 g/dL (Noblett et al 2021). While there is no consensus on a clinical Hb reference value for anemia, for the purpose of interpreting this study a value of < 9 g/d has been adopted to define clinical anemia diagnosis. Using clinical reference values of 9 g/dL, only 15 pigs in this experiment could be classified as anemic at weaning, suggesting that this population of pigs may not have been the best cohort to test the experimental treatments imposed. Anemia, by definition, is expressed as low Hb concentrations. This classification alone provides little insight into the mechanism that results in low Hb. There are a variety of different factors such as lack of vitamin B_12_ (megaloblastic anemia), insufficient production by bone marrow (aplastic anemia), and increased breakdown of healthy red blood cells (hemolytic anemia) that can all cause certain types of anemias that result in low red blood cell counts. The importance of other factors to aid in the diagnosis of each specific anemia is therefore crucial. For the diagnosis of IDA, ferritin is an essential component of the diagnosis, as it is a marker of iron storage and is commonly used in IDA diagnosis in human medicine, but not in pigs (Babaei et al 2017).

When ferritin concentrations are taken into consideration, concentrations were likely not low enough to indicate IDA as all treatments had ferritin concentrations above 30 ng/ml. Therefore, it is likely that the target pigs of this experiment were not observed. Alternatively, the prevalence of anemia within the population in this experiment was too low to accurately quantify the effects of additional iron supplementation on these anemic pigs, and this may be an indication that IDA is not as much of a concern as previously thought.

Moreover, the low weaning age of the pigs used in this experiment (20.2 days ± 0.07) is a possible factor relating to growth performance, as younger pigs may be subject to stress around weaning (Main et al 2004). Furthermore, the quantification of ferritin is becoming increasingly important in the diagnosis of IDA. Similar to Hb, there is poor understanding of clinical ferritin concentrations in pigs. In humans plasma ferritin concentrations between 30 and 45 ng/mL have been proposed for clinical IDA diagnosis (Ioannou et al 2007; Garcia-Casal et al 2021), but like Hb concentration to define anemia there is some debate, with some research suggesting 15 ng/mL is an appropriate threshold (Killip et al 2007). Furthermore, ferritin alone is not a reliable marker of IDA (Garcia-Casal et al 2021) and should be used in combination with Hb concentrations for an appropriate diagnosis (Gelaw et al 2019). Although interspecies differences are expected, these values correspond to the concentrations measured in pigs in this experiment (cohort average 34.5 ng/mL). These values are all derived from human medicine due to a lack of research around pig anemia, which is interesting because iron supplementation has been a routine practice for commercial pigs since the 1950s (Pond et al 1965). This further highlights the importance of quantifying anemia using more than just Hb concentration as a diagnostic tool. However, with current point-of-care measurements available, Hb is still the most practical marker as it can be done relatively quickly and without the need to send samples for further laboratory analysis.

The previously observed relationship between increased Hb and increased growth was not observed in this experiment. Several studies have shown that an injection on day 7 leads to increased growth performance (Chevalier et al 2023; Pierce et al 2024). Furthermore, it was highly surprising that there were no improvements in any growth parameters in the day 7 group, despite significant improvements in Hb concentrations. This leads to a possible conclusion that other factors that were not quantified in this experiment may have suppressed any increases in growth related to the day 7 injection. The weaning treatment did yield some trends for improved growth performance in the second week after weaning. However, it is unclear why this occurred, but further research into the growth effects of an additional injection at weaning is necessary, as there are other benefits other than improved growth, such as reduced handling of pigs as it is common for pigs to receive injections at weaning and handling already occurs at this time. One possible explanation for the improvements in growth is the changes in behavior in the weaning iron treatment group. The weaning iron treatment pigs spent more time resting than pigs that did not receive an injection, but no less time feeding. Moreover, BHB was significantly elevated in the Day 7 group, although below the threshold of 0.2 mmol/L for ketosis (Perri et al 2016), this likely indicates that a greater number of pigs in the day 7 treatment group had greater difficulty with the weaning transition than the weaning and control groups. This reduction of energy used in activity from days four to seven may have contributed to the trends in growth observed during the second week after weaning. Furthermore, the elevated HAMP concentrations in the weaning iron treatment group may have been significantly elevated, potentially impairing enteric iron absorption. However, this did not appear to affect Hb concentrations, as they did not differ significantly from those of the control pigs, and growth was improved rather than reduced.

In conclusion, an additional iron injection on day 7 was more successful than a weaning iron injection at improving Hb concentrations for 18 days post-weaning, by which time pigs have transitioned to receiving their iron requirements from feed. The weaning iron treatment did not affect Hb concentrations, but increased HAMP. While this may have consequences for enteric iron absorption, no negative effects were observed on ADG, with a trend for this to be improved in the second week after weaning and a significant reduction in the number of pigs removed from the experiment. The outcomes of this experiment support a greater survey to better understand the relationship between circulating Hb concentrations, which is the primary current method of identifying IDA in the pig herd and “true” anemia, which has a broader definition including parameters such as ferritin, and the effects of a weaning iron injection on potential ADG and health improvements. Correlating these parameters to production variables such as growth rates will allow for an improved understanding surrounding how IDA impacts pork production.

## Data Availability

The data is not publicly available.

## Abbreviations:

ADFI: average daily feed intake
ADG: average daily gain
BHB: beta-hydroxybutyrate
HAMP: hepcidin anti-microbial protein
FCE: feed conversion efficiency
Hb: hemoglobin
IDA: iron-deficient anemia
